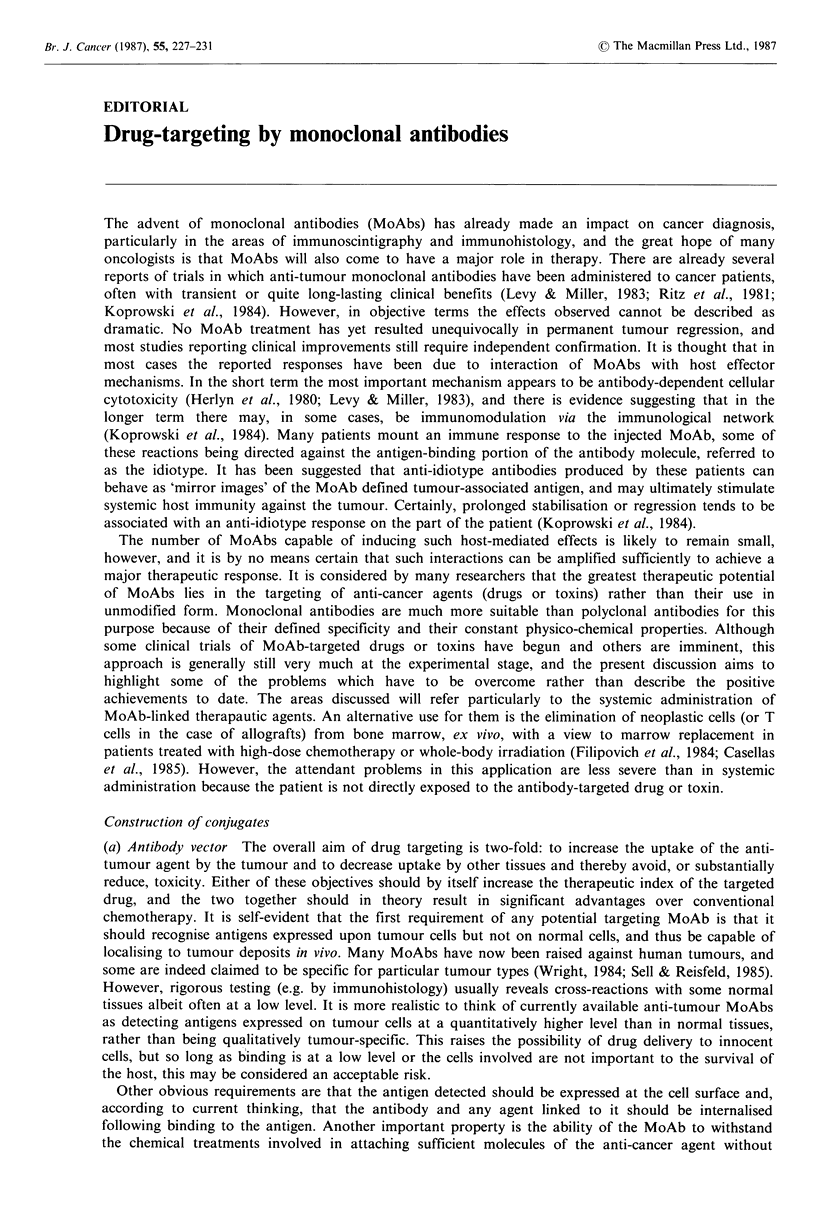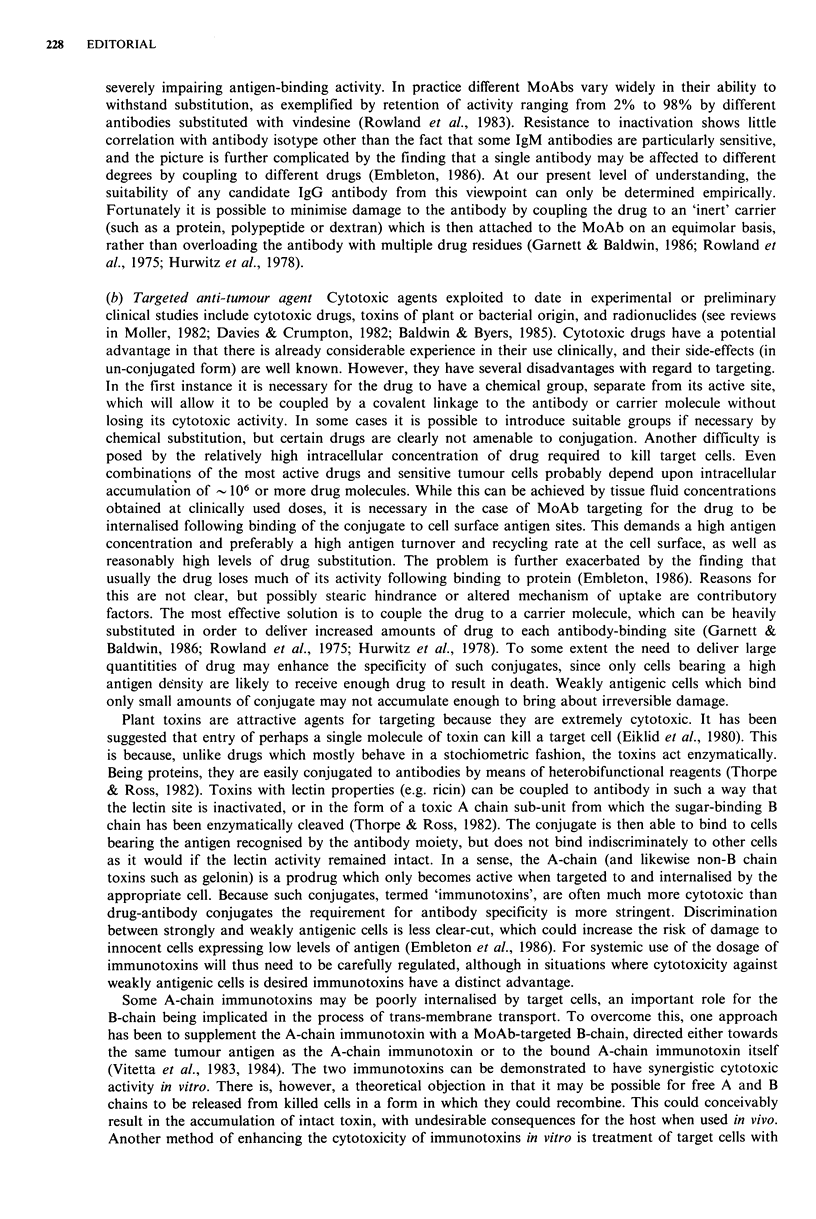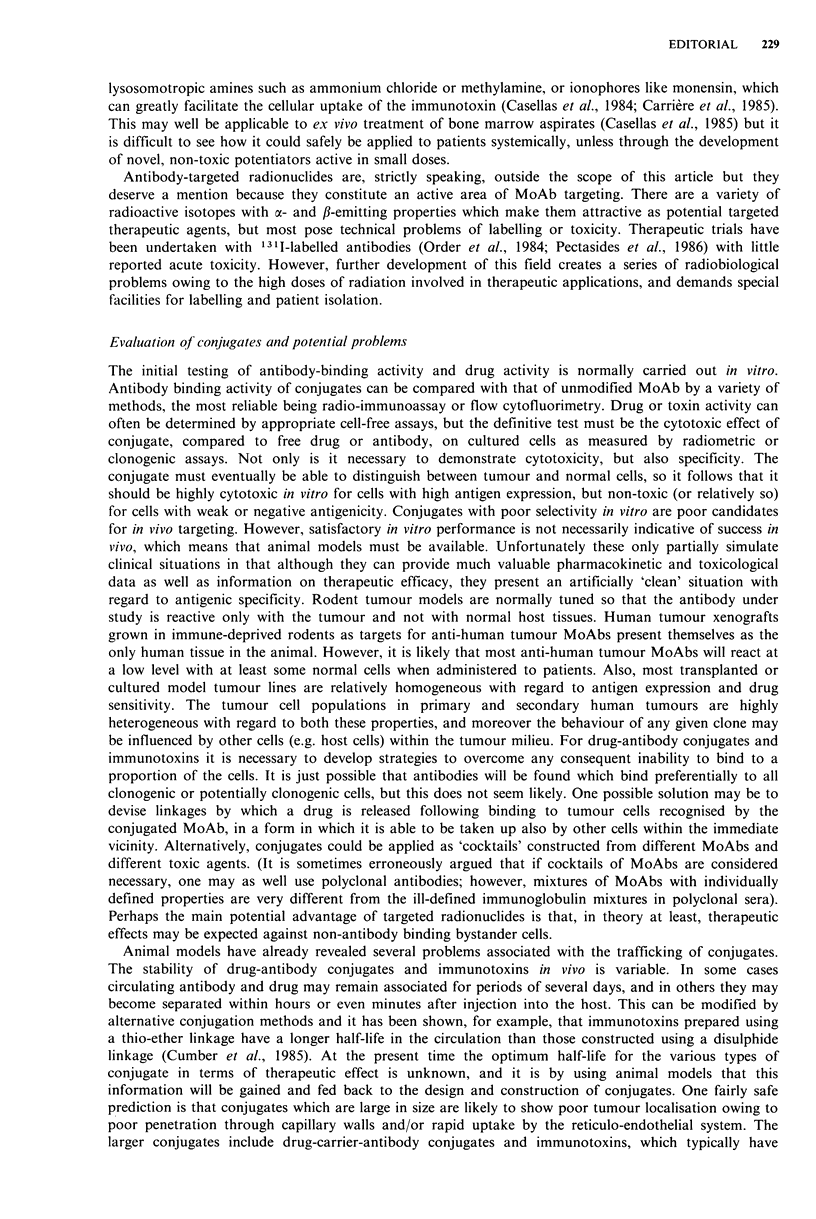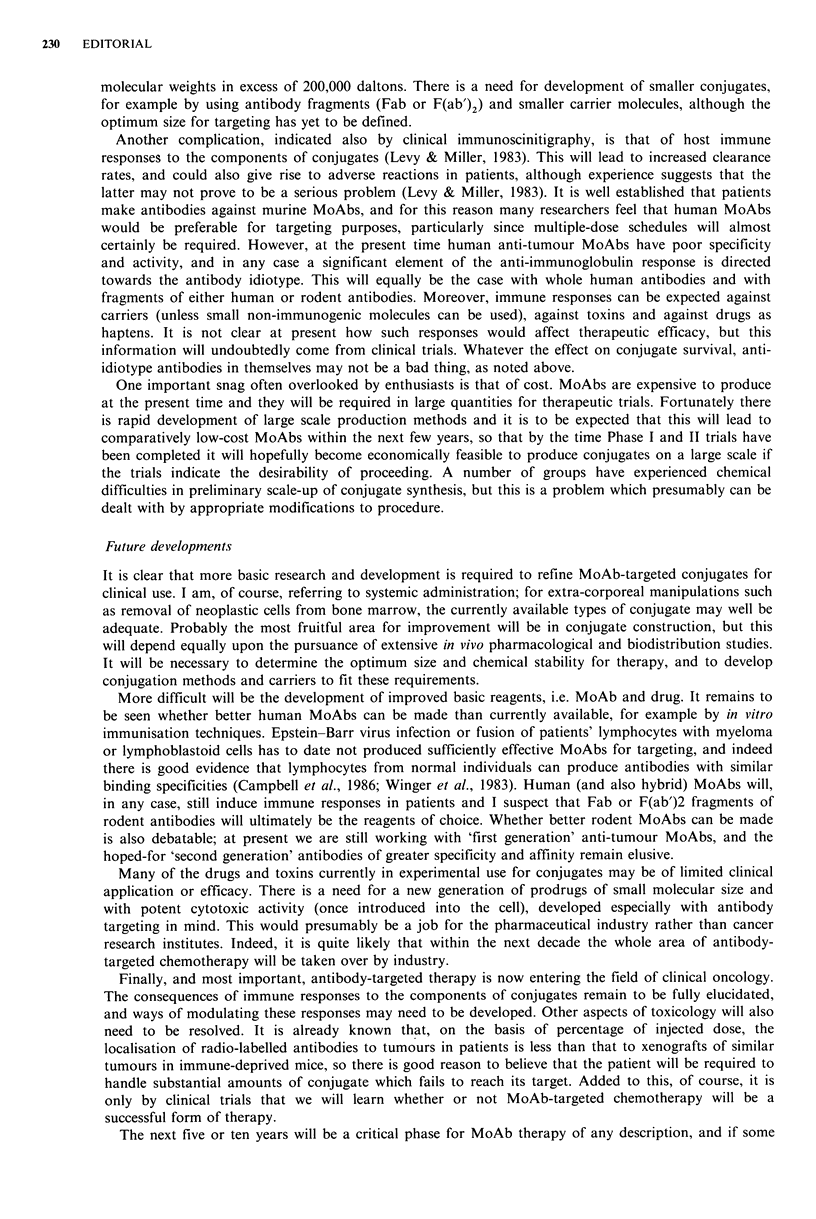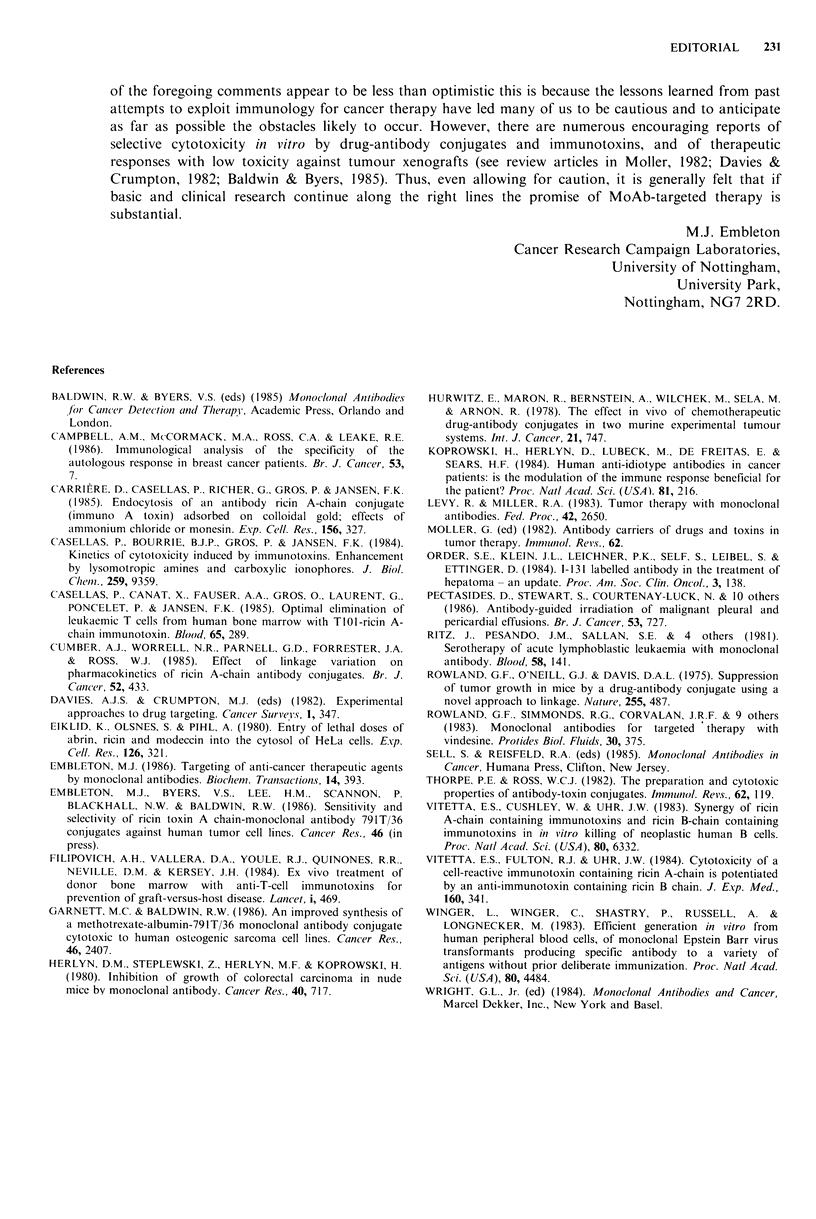# Drug-targeting by monoclonal antibodies.

**DOI:** 10.1038/bjc.1987.44

**Published:** 1987-03

**Authors:** M. J. Embleton


Br. J. Cancer (1987), 55, 227–231 © The Macmillan Press Ltd., 1987

EDITORIAL

### Drug-targeting by monoclonal antibodies

The advent of monoclonal antibodies (MoAbs) has already made an impact on cancer diagnosis, particularly in the areas of immunoscintigraphy and immunohistology, and the great hope of many oncologists is that MoAbs will also come to have a major role in therapy. There are already several reports of trials in which anti-tumour monoclonal antibodies have been administered to cancer patients, often with transient or quite long-lasting clinical benefits (Levy & Miller, 1983; Ritz *et al.*, 1981; Koprowski *et al.*, 1984). However, in objective terms the effects observed cannot be described as dramatic. No MoAb treatment has yet resulted unequivocally in permanent tumour regression, and most studies reporting clinical improvements still require independent confirmation. It is thought that in most cases the reported responses have been due to interaction of MoAbs with host effector mechanisms. In the short term the most important mechanism appears to be antibody-dependent cellular cytotoxicity (Herlyn *et al.*, 1980; Levy & Miller, 1983), and there is evidence suggesting that in the longer term there may, in some cases, be immunomodulation *via* the immunological network (Koprowski *et al.*, 1984). Many patients mount an immune response to the injected MoAb, some of these reactions being directed against the antigen-binding portion of the antibody molecule, referred to as the idiotype. It has been suggested that anti-idiotype antibodies produced by these patients can behave as 'mirror images' of the MoAb defined tumour-associated antigen, and may ultimately stimulate systemic host immunity against the tumour. Certainly, prolonged stabilisation or regression tends to be associated with an anti-idiotype response on the part of the patient (Koprowski *et al.*, 1984).

The number of MoAbs capable of inducing such host-mediated effects is likely to remain small, however, and it is by no means certain that such interactions can be amplified sufficiently to achieve a major therapeutic response. It is considered by many researchers that the greatest therapeutic potential of MoAbs lies in the targeting of anti-cancer agents (drugs or toxins) rather than their use in unmodified form. Monoclonal antibodies are much more suitable than polyclonal antibodies for this purpose because of their defined specificity and their constant physico-chemical properties. Although some clinical trials of MoAb-targeted drugs or toxins have begun and others are imminent, this approach is generally still very much at the experimental stage, and the present discussion aims to highlight some of the problems which have to be overcome rather than describe the positive achievements to date. The areas discussed will refer particularly to the systemic administration of MoAb-linked therapautic agents. An alternative use for them is the elimination of neoplastic cells (or T cells in the case of allografts) from bone marrow, *ex vivo*, with a view to marrow replacement in patients treated with high-dose chemotherapy or whole-body irradiation (Filipovich *et al.*, 1984; Casellas *et al.*, 1985). However, the attendant problems in this application are less severe than in systemic administration because the patient is not directly exposed to the antibody-targeted drug or toxin.

*Construction of conjugates*

(*a*) *Antibody vector* The overall aim of drug targeting is two-fold: to increase the uptake of the anti-tumour agent by the tumour and to decrease uptake by other tissues and thereby avoid, or substantially reduce, toxicity. Either of these objectives should by itself increase the therapeutic index of the targeted drug, and the two together should in theory result in significant advantages over conventional chemotherapy. It is self-evident that the first requirement of any potential targeting MoAb is that it should recognise antigens expressed upon tumour cells but not on normal cells, and thus be capable of localising to tumour deposits *in vivo*. Many MoAbs have now been raised against human tumours, and some are indeed claimed to be specific for particular tumour types (Wright, 1984; Sell & Reisfeld, 1985). However, rigorous testing (e.g. by immunohistology) usually reveals cross-reactions with some normal tissues albeit often at a low level. It is more realistic to think of currently available anti-tumour MoAbs as detecting antigens expressed on tumour cells at a quantitatively higher level than in normal tissues, rather than being qualitatively tumour-specific. This raises the possibility of drug delivery to innocent cells, but so long as binding is at a low level or the cells involved are not important to the survival of the host, this may be considered an acceptable risk.

Other obvious requirements are that the antigen detected should be expressed at the cell surface and, according to current thinking, that the antibody and any agent linked to it should be internalised following binding to the antigen. Another important property is the ability of the MoAb to withstand the chemical treatments involved in attaching sufficient molecules of the anti-cancer agent without severely impairing antigen-binding activity. In practice different MoAbs vary widely in their ability to withstand substitution, as exemplified by retention of activity ranging from 2% to 98% by different antibodies substituted with vindesine (Rowland *et al.*, 1983). Resistance to inactivation shows little correlation with antibody isotype other than the fact that some IgM antibodies are particularly sensitive, and the picture is further complicated by the finding that a single antibody may be affected to different degrees by coupling to different drugs (Embleton, 1986). At our present level of understanding, the suitability of any candidate IgG antibody from this viewpoint can only be determined empirically. Fortunately it is possible to minimise damage to the antibody by coupling the drug to an 'inert' carrier (such as a protein, polypeptide or dextran) which is then attached to the MoAb on an equimolar basis, rather than overloading the antibody with multiple drug residues (Garnett & Baldwin, 1986; Rowland *et al.*, 1975; Hurwitz *et al.*, 1978).

(*b*) *Targeted anti-tumour agent* Cytotoxic agents exploited to date in experimental or preliminary clinical studies include cytotoxic drugs, toxins of plant or bacterial origin, and radionuclides (see reviews in Moller, 1982; Davies & Crumpton, 1982; Baldwin & Byers, 1985). Cytotoxic drugs have a potential advantage in that there is already considerable experience in their use clinically, and their side-effects (in un-conjugated form) are well known. However, they have several disadvantages with regard to targeting. In the first instance it is necessary for the drug to have a chemical group, separate from its active site, which will allow it to be coupled by a covalent linkage to the antibody or carrier molecule without losing its cytotoxic activity. In some cases it is possible to introduce suitable groups if necessary by chemical substitution, but certain drugs are clearly not amenable to conjugation. Another difficulty is posed by the relatively high intracellular concentration of drug required to kill target cells. Even combinations of the most active drugs and sensitive tumour cells probably depend upon intracellular accumulation of $\sim 10^6$ or more drug molecules. While this can be achieved by tissue fluid concentrations obtained at clinically used doses, it is necessary in the case of MoAb targeting for the drug to be internalised following binding of the conjugate to cell surface antigen sites. This demands a high antigen concentration and preferably a high antigen turnover and recycling rate at the cell surface, as well as reasonably high levels of drug substitution. The problem is further exacerbated by the finding that usually the drug loses much of its activity following binding to protein (Embleton, 1986). Reasons for this are not clear, but possibly stearic hindrance or altered mechanism of uptake are contributory factors. The most effective solution is to couple the drug to a carrier molecule, which can be heavily substituted in order to deliver increased amounts of drug to each antibody-binding site (Garnett & Baldwin, 1986; Rowland *et al.*, 1975; Hurwitz *et al.*, 1978). To some extent the need to deliver large quantitities of drug may enhance the specificity of such conjugates, since only cells bearing a high antigen density are likely to receive enough drug to result in death. Weakly antigenic cells which bind only small amounts of conjugate may not accumulate enough to bring about irreversible damage.

Plant toxins are attractive agents for targeting because they are extremely cytotoxic. It has been suggested that entry of perhaps a single molecule of toxin can kill a target cell (Eiklid *et al.*, 1980). This is because, unlike drugs which mostly behave in a stochiometric fashion, the toxins act enzymatically. Being proteins, they are easily conjugated to antibodies by means of heterobifunctional reagents (Thorpe & Ross, 1982). Toxins with lectin properties (e.g. ricin) can be coupled to antibody in such a way that the lectin site is inactivated, or in the form of a toxic A chain sub-unit from which the sugar-binding B chain has been enzymatically cleaved (Thorpe & Ross, 1982). The conjugate is then able to bind to cells bearing the antigen recognised by the antibody moiety, but does not bind indiscriminately to other cells as it would if the lectin activity remained intact. In a sense, the A-chain (and likewise non-B chain toxins such as gelonin) is a prodrug which only becomes active when targeted to and internalised by the appropriate cell. Because such conjugates, termed 'immunotoxins', are often much more cytotoxic than drug-antibody conjugates the requirement for antibody specificity is more stringent. Discrimination between strongly and weakly antigenic cells is less clear-cut, which could increase the risk of damage to innocent cells expressing low levels of antigen (Embleton *et al.*, 1986). For systemic use of the dosage of immunotoxins will thus need to be carefully regulated, although in situations where cytotoxicity against weakly antigenic cells is desired immunotoxins have a distinct advantage.

Some A-chain immunotoxins may be poorly internalised by target cells, an important role for the B-chain being implicated in the process of trans-membrane transport. To overcome this, one approach has been to supplement the A-chain immunotoxin with a MoAb-targeted B-chain, directed either towards the same tumour antigen as the A-chain immunotoxin or to the bound A-chain immunotoxin itself (Vitetta *et al.*, 1983, 1984). The two immunotoxins can be demonstrated to have synergistic cytotoxic activity *in vitro*. There is, however, a theoretical objection in that it may be possible for free A and B chains to be released from killed cells in a form in which they could recombine. This could conceivably result in the accumulation of intact toxin, with undesirable consequences for the host when used *in vivo*. Another method of enhancing the cytotoxicity of immunotoxins *in vitro* is treatment of target cells with lysosomotropic amines such as ammonium chloride or methylamine, or ionophores like monensin, which can greatly facilitate the cellular uptake of the immunotoxin (Casellas *et al.*, 1984; Carrière *et al.*, 1985). This may well be applicable to *ex vivo* treatment of bone marrow aspirates (Casellas *et al.*, 1985) but it is difficult to see how it could safely be applied to patients systemically, unless through the development of novel, non-toxic potentiators active in small doses.

Antibody-targeted radionuclides are, strictly speaking, outside the scope of this article but they deserve a mention because they constitute an active area of MoAb targeting. There are a variety of radioactive isotopes with $\alpha$- and $\beta$-emitting properties which make them attractive as potential targeted therapeutic agents, but most pose technical problems of labelling or toxicity. Therapeutic trials have been undertaken with $^{131}I$-labelled antibodies (Order *et al.*, 1984; Pectasides *et al.*, 1986) with little reported acute toxicity. However, further development of this field creates a series of radiobiological problems owing to the high doses of radiation involved in therapeutic applications, and demands special facilities for labelling and patient isolation.

*Evaluation of conjugates and potential problems*

The initial testing of antibody-binding activity and drug activity is normally carried out *in vitro*. Antibody binding activity of conjugates can be compared with that of unmodified MoAb by a variety of methods, the most reliable being radio-immunoassay or flow cytofluorimetry. Drug or toxin activity can often be determined by appropriate cell-free assays, but the definitive test must be the cytotoxic effect of conjugate, compared to free drug or antibody, on cultured cells as measured by radiometric or clonogenic assays. Not only is it necessary to demonstrate cytotoxicity, but also specificity. The conjugate must eventually be able to distinguish between tumour and normal cells, so it follows that it should be highly cytotoxic *in vitro* for cells with high antigen expression, but non-toxic (or relatively so) for cells with weak or negative antigenicity. Conjugates with poor selectivity *in vitro* are poor candidates for *in vivo* targeting. However, satisfactory *in vitro* performance is not necessarily indicative of success *in vivo*, which means that animal models must be available. Unfortunately these only partially simulate clinical situations in that although they can provide much valuable pharmacokinetic and toxicological data as well as information on therapeutic efficacy, they present an artificially 'clean' situation with regard to antigenic specificity. Rodent tumour models are normally tuned so that the antibody under study is reactive only with the tumour and not with normal host tissues. Human tumour xenografts grown in immune-deprived rodents as targets for anti-human tumour MoAbs present themselves as the only human tissue in the animal. However, it is likely that most anti-human tumour MoAbs will react at a low level with at least some normal cells when administered to patients. Also, most transplanted or cultured model tumour lines are relatively homogeneous with regard to antigen expression and drug sensitivity. The tumour cell populations in primary and secondary human tumours are highly heterogeneous with regard to both these properties, and moreover the behaviour of any given clone may be influenced by other cells (e.g. host cells) within the tumour milieu. For drug-antibody conjugates and immunotoxins it is necessary to develop strategies to overcome any consequent inability to bind to a proportion of the cells. It is just possible that antibodies will be found which bind preferentially to all clonogenic or potentially clonogenic cells, but this does not seem likely. One possible solution may be to devise linkages by which a drug is released following binding to tumour cells recognised by the conjugated MoAb, in a form in which it is able to be taken up also by other cells within the immediate vicinity. Alternatively, conjugates could be applied as 'cocktails' constructed from different MoAbs and different toxic agents. (It is sometimes erroneously argued that if cocktails of MoAbs are considered necessary, one may as well use polyclonal antibodies; however, mixtures of MoAbs with individually defined properties are very different from the ill-defined immunoglobulin mixtures in polyclonal sera). Perhaps the main potential advantage of targeted radionuclides is that, in theory at least, therapeutic effects may be expected against non-antibody binding bystander cells.

Animal models have already revealed several problems associated with the trafficking of conjugates. The stability of drug-antibody conjugates and immunotoxins *in vivo* is variable. In some cases circulating antibody and drug may remain associated for periods of several days, and in others they may become separated within hours or even minutes after injection into the host. This can be modified by alternative conjugation methods and it has been shown, for example, that immunotoxins prepared using a thio-ether linkage have a longer half-life in the circulation than those constructed using a disulphide linkage (Cumber *et al.*, 1985). At the present time the optimum half-life for the various types of conjugate in terms of therapeutic effect is unknown, and it is by using animal models that this information will be gained and fed back to the design and construction of conjugates. One fairly safe prediction is that conjugates which are large in size are likely to show poor tumour localisation owing to poor penetration through capillary walls and/or rapid uptake by the reticulo-endothelial system. The larger conjugates include drug-carrier-antibody conjugates and immunotoxins, which typically have molecular weights in excess of 200,000 daltons. There is a need for development of smaller conjugates, for example by using antibody fragments (Fab or $F(ab')_2$) and smaller carrier molecules, although the optimum size for targeting has yet to be defined.

Another complication, indicated also by clinical immunoscinitigraphy, is that of host immune responses to the components of conjugates (Levy & Miller, 1983). This will lead to increased clearance rates, and could also give rise to adverse reactions in patients, although experience suggests that the latter may not prove to be a serious problem (Levy & Miller, 1983). It is well established that patients make antibodies against murine MoAbs, and for this reason many researchers feel that human MoAbs would be preferable for targeting purposes, particularly since multiple-dose schedules will almost certainly be required. However, at the present time human anti-tumour MoAbs have poor specificity and activity, and in any case a significant element of the anti-immunoglobulin response is directed towards the antibody idiotype. This will equally be the case with whole human antibodies and with fragments of either human or rodent antibodies. Moreover, immune responses can be expected against carriers (unless small non-immunogenic molecules can be used), against toxins and against drugs as haptens. It is not clear at present how such responses would affect therapeutic efficacy, but this information will undoubtedly come from clinical trials. Whatever the effect on conjugate survival, anti-idiotype antibodies in themselves may not be a bad thing, as noted above.

One important snag often overlooked by enthusiasts is that of cost. MoAbs are expensive to produce at the present time and they will be required in large quantities for therapeutic trials. Fortunately there is rapid development of large scale production methods and it is to be expected that this will lead to comparatively low-cost MoAbs within the next few years, so that by the time Phase I and II trials have been completed it will hopefully become economically feasible to produce conjugates on a large scale if the trials indicate the desirability of proceeding. A number of groups have experienced chemical difficulties in preliminary scale-up of conjugate synthesis, but this is a problem which presumably can be dealt with by appropriate modifications to procedure.

### *Future developments*

It is clear that more basic research and development is required to refine MoAb-targeted conjugates for clinical use. I am, of course, referring to systemic administration; for extra-corporeal manipulations such as removal of neoplastic cells from bone marrow, the currently available types of conjugate may well be adequate. Probably the most fruitful area for improvement will be in conjugate construction, but this will depend equally upon the pursuance of extensive *in vivo* pharmacological and biodistribution studies. It will be necessary to determine the optimum size and chemical stability for therapy, and to develop conjugation methods and carriers to fit these requirements.

More difficult will be the development of improved basic reagents, i.e. MoAb and drug. It remains to be seen whether better human MoAbs can be made than currently available, for example by *in vitro* immunisation techniques. Epstein–Barr virus infection or fusion of patients' lymphocytes with myeloma or lymphoblastoid cells has to date not produced sufficiently effective MoAbs for targeting, and indeed there is good evidence that lymphocytes from normal individuals can produce antibodies with similar binding specificities (Campbell *et al.*, 1986; Winger *et al.*, 1983). Human (and also hybrid) MoAbs will, in any case, still induce immune responses in patients and I suspect that Fab or F(ab')2 fragments of rodent antibodies will ultimately be the reagents of choice. Whether better rodent MoAbs can be made is also debatable; at present we are still working with 'first generation' anti-tumour MoAbs, and the hoped-for 'second generation' antibodies of greater specificity and affinity remain elusive.

Many of the drugs and toxins currently in experimental use for conjugates may be of limited clinical application or efficacy. There is a need for a new generation of prodrugs of small molecular size and with potent cytotoxic activity (once introduced into the cell), developed especially with antibody targeting in mind. This would presumably be a job for the pharmaceutical industry rather than cancer research institutes. Indeed, it is quite likely that within the next decade the whole area of antibody-targeted chemotherapy will be taken over by industry.

Finally, and most important, antibody-targeted therapy is now entering the field of clinical oncology. The consequences of immune responses to the components of conjugates remain to be fully elucidated, and ways of modulating these responses may need to be developed. Other aspects of toxicology will also need to be resolved. It is already known that, on the basis of percentage of injected dose, the localisation of radio-labelled antibodies to tumours in patients is less than that to xenografts of similar tumours in immune-deprived mice, so there is good reason to believe that the patient will be required to handle substantial amounts of conjugate which fails to reach its target. Added to this, of course, it is only by clinical trials that we will learn whether or not MoAb-targeted chemotherapy will be a successful form of therapy.

The next five or ten years will be a critical phase for MoAb therapy of any description, and if some of the foregoing comments appear to be less than optimistic this is because the lessons learned from past attempts to exploit immunology for cancer therapy have led many of us to be cautious and to anticipate as far as possible the obstacles likely to occur. However, there are numerous encouraging reports of selective cytotoxicity *in vitro* by drug-antibody conjugates and immunotoxins, and of therapeutic responses with low toxicity against tumour xenografts (see review articles in Moller, 1982; Davies & Crumpton, 1982; Baldwin & Byers, 1985). Thus, even allowing for caution, it is generally felt that if basic and clinical research continue along the right lines the promise of MoAb-targeted therapy is substantial.

M.J. Embleton
Cancer Research Campaign Laboratories,
University of Nottingham,
University Park,
Nottingham, NG7 2RD.

### References

BALDWIN, R.W. & BYERS, V.S. (eds) (1985) *Monoclonal Antibodies for Cancer Detection and Therapy*, Academic Press, Orlando and London.

CAMPBELL, A.M., McCORMACK, M.A., ROSS, C.A. & LEAKE, R.E. (1986). Immunological analysis of the specificity of the autologous response in breast cancer patients. *Br. J. Cancer*, **53**, 7.

CARRIÈRE, D., CASELLAS, P., RICHER, G., GROS, P. & JANSEN, F.K. (1985). Endocytosis of an antibody ricin A-chain conjugate (immuno A toxin) adsorbed on colloidal gold; effects of ammonium chloride or monesin. *Exp. Cell. Res.*, **156**, 327.

CASELLAS, P., BOURRIE, B.J.P., GROS, P. & JANSEN, F.K. (1984). Kinetics of cytotoxicity induced by immunotoxins. Enhancement by lysomotropic amines and carboxylic ionophores. *J. Biol. Chem.*, **259**, 9359.

CASELLAS, P., CANAT, X., FAUSER, A.A., GROS, O., LAURENT, G., PONCELET, P. & JANSEN, F.K. (1985). Optimal elimination of leukaemic T cells from human bone marrow with T101-ricin A-chain immunotoxin. *Blood*, **65**, 289.

CUMBER, A.J., WORRELL, N.R., PARNELL, G.D., FORRESTER, J.A. & ROSS, W.J. (1985). Effect of linkage variation on pharmacokinetics of ricin A-chain antibody conjugates. *Br. J. Cancer*, **52**, 433.

DAVIES, A.J.S. & CRUMPTON, M.J. (eds) (1982). Experimental approaches to drug targeting. *Cancer Surveys*, **1**, 347.

EIKLID, K., OLSNES, S. & PIHL, A. (1980). Entry of lethal doses of abrin, ricin and modeccin into the cytosol of HeLa cells. *Exp. Cell. Res.*, **126**, 321.

EMBLETON, M.J. (1986). Targeting of anti-cancer therapeutic agents by monoclonal antibodies. *Biochem. Transactions*, **14**, 393.

EMBLETON, M.J., BYERS, V.S., LEE, H.M., SCANNON, P. BLACKHALL, N.W. & BALDWIN, R.W. (1986). Sensitivity and selectivity of ricin toxin A chain-monoclonal antibody 791T/36 conjugates against human tumor cell lines. *Cancer Res.*, **46** (in press).

FILIPOVICH, A.H., VALLERA, D.A., YOULE, R.J., QUINONES, R.R., NEVILLE, D.M. & KERSEY, J.H. (1984). Ex vivo treatment of donor bone marrow with anti-T-cell immunotoxins for prevention of graft-versus-host disease. *Lancet*, **i**, 469.

GARNETT, M.C. & BALDWIN, R.W. (1986). An improved synthesis of a methotrexate-albumin-791T/36 monoclonal antibody conjugate cytotoxic to human osteogenic sarcoma cell lines. *Cancer Res.*, **46**, 2407.

HERLYN, D.M., STEPLEWSKI, Z., HERLYN, M.F. & KOPROWSKI, H. (1980). Inhibition of growth of colorectal carcinoma in nude mice by monoclonal antibody. *Cancer Res.*, **40**, 717.

HURWITZ, E., MARON, R., BERNSTEIN, A., WILCHEK, M., SELA, M. & ARNON, R. (1978). The effect in vivo of chemotherapeutic drug-antibody conjugates in two murine experimental tumour systems. *Int. J. Cancer*, **21**, 747.

KOPROWSKI, H., HERLYN, D., LUBECK, M., DE FREITAS, E. & SEARS, H.F. (1984). Human anti-idiotype antibodies in cancer patients: is the modulation of the immune response beneficial for the patient? *Proc. Natl Acad. Sci. (USA)*. **81**, 216.

LEVY, R. & MILLER, R.A. (1983). Tumor therapy with monoclonal antibodies. *Fed. Proc.*, **42**, 2650.

MOLLER, G. (ed) (1982). Antibody carriers of drugs and toxins in tumor therapy. *Immunol. Revs.*, **62**.

ORDER, S.E., KLEIN, J.L., LEICHNER, P.K., SELF, S., LEIBEL, S. & ETTINGER, D. (1984). I-131 labelled antibody in the treatment of hepatoma – an update. *Proc. Am. Soc. Clin. Oncol.*, **3**, 138.

PECTASIDES, D., STEWART, S., COURTENAY-LUCK, N. & 10 others (1986). Antibody-guided irradiation of malignant pleural and pericardial effusions. *Br. J. Cancer*, **53**, 727.

RITZ, J., PESANDO, J.M., SALLAN, S.E. & 4 others (1981). Serotherapy of acute lymphoblastic leukaemia with monoclonal antibody. *Blood*, **58**, 141.

ROWLAND, G.F., O'NEILL, G.J. & DAVIS, D.A.L. (1975). Suppression of tumor growth in mice by a drug-antibody conjugate using a novel approach to linkage. *Nature*, **255**, 487.

ROWLAND, G.F., SIMMONDS, R.G., CORVALAN, J.R.F. & 9 others (1983). Monoclonal antibodies for targeted therapy with vindesine. *Protides Biol. Fluids*, **30**, 375.

SELL, S. & REISFELD, R.A. (eds) (1985). *Monoclonal Antibodies in Cancer*, Humana Press, Clifton, New Jersey.

THORPE, P.E. & ROSS, W.C.J. (1982). The preparation and cytotoxic properties of antibody-toxin conjugates. *Immunol. Revs.*, **62**, 119.

VITETTA, E.S., CUSHLEY, W. & UHR, J.W. (1983). Synergy of ricin A-chain containing immunotoxins and ricin B-chain containing immunotoxins in *in vitro* killing of neoplastic human B cells. *Proc. Natl Acad. Sci. (USA)*, **80**, 6332.

VITETTA, E.S., FULTON, R.J. & UHR, J.W. (1984). Cytotoxicity of a cell-reactive immunotoxin containing ricin A-chain is potentiated by an anti-immunotoxin containing ricin B chain. *J. Exp. Med.*, **160**, 341.

WINGER, L., WINGER, C., SHASTRY, P., RUSSELL, A. & LONGNECKER, M. (1983). Efficient generation *in vitro* from human peripheral blood cells, of monoclonal Epstein Barr virus transformants producing specific antibody to a variety of antigens without prior deliberate immunization. *Proc. Natl Acad. Sci. (USA)*, **80**, 4484.

WRIGHT, G.L., Jr. (ed) (1984). *Monoclonal Antibodies and Cancer*, Marcel Dekker, Inc., New York and Basel.